# Do Gender-Related Stereotypes Affect Spatial Performance? Exploring When, How and to Whom Using a Chronometric Two-Choice Mental Rotation Task

**DOI:** 10.3389/fpsyg.2018.01261

**Published:** 2018-07-24

**Authors:** Carla Sanchis-Segura, Naiara Aguirre, Álvaro J. Cruz-Gómez, Noemí Solozano, Cristina Forn

**Affiliations:** Departamento de Psicologia Básica, Clínica y Psicobiología, Facultad de Ciencias de la Salud, Universitat Jaume I, Castellón, Spain

**Keywords:** gender stereotypes, stereotype threat, mental rotation, implicit association test, STEM

## Abstract

It is a common belief that males have superior visuospatial abilities and that differences in this and other cognitive domains (e.g., math) contribute to the reduced interest and low representation of girls and women in STEM education and professions. However, previous studies show that gender-related implicit associations and explicit beliefs, as well as situational variables, might affect cognitive performance in those gender-stereotyped domains and produce between-gender spurious differences. Therefore, the present study aimed to provide information on when, how and who might be affected by the situational reactivation of stereotypic gender-science beliefs/associations while performing a 3D mental rotation chronometric task (3DMRT). More specifically, we assessed the explicit beliefs and implicit associations (by the Implicit Association Test) held by female and male students of humanities and STEM majors and compared their performance in a 3DMRT after receiving stereotype- congruent, incongruent and nullifying experimental instructions. Our results show that implicit stereotypic gender-science associations correlate with 3DMRT performance in both females and males, but that inter-gender differences emerge only under stereotype-reactivating conditions. We also found that changes in self-confidence mediate these instructions’ effects and that academic specialization moderates them, hence promoting 3DMRT performance differences between male and female humanities, but not STEM, students. Taken together, these observations suggest that the common statement “males have superior mental rotation abilities” simplifies a much more complex reality and might promote stereotypes which, in turn, might induce artefactual performance differences between females and males in such tasks.

## Introduction

Although in elementary, middle, and high school, girls and boys take math and science courses in roughly equal numbers, only around 20 percent of STEM graduates are women, a number that declines even further in the workplace (Hill et al., 2010; European Commission, 2016). Because STEM related careers are expected to grow faster than the average rate for all occupations (National Science Board, 2010) and are among the best paid jobs (National Association of Colleges and Employers, 2009; European Commission, 2016), the underrepresentation of women in STEM studies severely increases the risk of exclusion and precarization in their future incorporation into the labor market. Yet, this is not only a problem for women. The absence of women from STEM education and careers is a waste of talent for those fields (European Commission, 2014; Norland et al., 2016) and also an economic cost for society as a whole. Indeed, it has been estimated that closing the gender gap in the STEM field would increase the EU GDP per capita by 0.7–0.9% in 2030 and by 2.2–3.0% in 2050 (Maceira, 2017). Accordingly, the gender segregation that characterizes the STEM field at the educational and professional level is seen with increasing social and institutional concern (Hill et al., 2010; European Commission, 2016).

The underrepresentation of women in STEM studies and professions has been traditionally considered a consequence of an innate higher proficiency of males in math and visuospatial abilities (Benbow et al., 2000; Baron-Cohen, 2004). Popularized through expressions such as “math is hard for girls” (Barbie, 1994, see Ben-Zeev et al., 2005) or “women cannot read maps” (Pease and Pease, 2004), the notion that males excel over females in these cognitive domains has become a widely shared social belief. However, scientific evidence does not support these claims and presents a much more complex reality (Ceci et al., 2009; Wang and Degol, 2013).

Thus, although older studies regularly identified a males’ advantage in math performance (Glennon and Callahan, 1968; Maccoby and Jacklin, 1974; Benbow and Stanley, 1980, 1983), more recent large-sample studies and metaanalyses have revealed that gender differences in mathematics achievement tend to be inconsistent and small (*d* = 0.05, Lindberg et al., 2010; *d* = 0.06; Voyer and Voyer, 2014). Moreover, the size and even the direction of average gender differences in math performance widely varies among countries (*d*s ranging -0.42 to 0.40, Else-Quest et al., 2010; OECD, 2016) and they are correlated to national gender equity indexes (Reilly, 2012). Similarly, the proportion of females and males scoring at the 95th or 99th percentiles also differ among countries (Guiso et al., 2008; Machin and Pekkarinen, 2008) and they are highly correlated to national gender equality indexes, (Guiso et al., 2008; Hyde and Mertz, 2009). Finally, theoretical models demonstrate that the number of women in STEM studies and professions is substantially lower than that predicted from their math performance (Hyde and Mertz, 2009). Taken together, these and other data (reviewed in Spelke, 2005; Halpern et al., 2007; Ceci et al., 2009; Wang and Degol, 2013) strongly argue against the notion that males have innate or “hard-wired” superior math abilities that could account for the underrepresentation of women in STEM studies and occupations.

On the other hand, spatial ability is the cognitive domain in which differences between males and females are most commonly replicated and reported (Voyer et al., 1995; Hyde, 2014). Among the tasks in which such differences are observed, mental rotation tasks (MRT) produce the largest effects (Linn and Petersen, 1985; Halpern, 2013), which meta-analyses and large-sample studies have estimated as being medium to large (Linn and Petersen, 1985; Voyer et al., 1995; Peters et al., 2007; Hyde, 2014). Conversely to what has been observed for math, gender differences in MRT are observed in all countries (Silverman et al., 2007) and their size do not seem to have declined over time (Masters and Sanders, 1993).

Given their high replicability, males-females’ differences in MRT performance have been traditionally regarded as “sex differences” in visuospatial competence that arise from brain specializations imposed by the organizing actions of testosterone during prenatal development (Grimshaw et al., 1995; Baron-Cohen, 2004; Kempel et al., 2005; Peters et al., 2007; Vuoksimaa et al., 2010) and/or from the sexual division of labor in human early evolutionary history (Silverman and Eals, 1992; Silverman et al., 2007). However, despite its popularity both inside and outside the scientific realm, the empirical evidence that supports these views is far from conclusive (Fausto-Sterling, 2003; Jones et al., 2003; Jordan-Young, 2010). Indeed, there is a poor correlation between visuospatial abilities and the indirect indices of prenatal testosterone exposure (Puts et al., 2008) and the “sex differences” regularly observed in this cognitive domain are moderated by subjects’ age (Geiser et al., 2008; Titze et al., 2010), experience and training (Uttal et al., 2012) as well as by task-related factors [e.g., time constrains (Voyer, 2011; Maeda and Yoon, 2013; kinds of stimuli (Alexander and Evardone, 2008; Ruthsatz et al., 2017)]. Furthermore, the biological and socio-cultural factors traditionally assigned to sex and gender are irremediably entangled and, in practice, it is not possible to separate their relative contribution to males and females’ behaviors as they form a complex set of intertwined influences, referred to as sex/gender (Fausto-Sterling, 2003; Kaiser et al., 2009; Springer et al., 2012). Accordingly, the study of behavioral and cognitive similarities, and the differences between females and males, require more complex and integrative formulations than those provided by traditional categorical divides (e.g., male *vs.* female; biological *vs.* social, etc.), and should incorporate the interactions among predisposing, experiential and situational variables (Jordan-Young and Rumiati, 2012; Springer et al., 2012; Rippon et al., 2014).

In line with this, accumulated evidence indicates that factors traditionally assigned to “gender” might boost the differences in MRT performance ordinarily attributed to “sex.” Indeed, it is well known that stereotypic beliefs about cognitive female-male differences can exert long-term effects on the acquisition of both interests and skills (Eccles, 1987; Bussey and Bandura, 1999), but may also have more immediate effects by affecting performance when situationally activated. Thus ever since childhood, self- or others’ endorsement of commonly held stereotypic beliefs and implicit associations about genders (e.g., “science-male”; Nosek et al., 2002) reduce female performance in cognitive domains culturally viewed as “masculine” (e.g., math; Ambady et al., 2001; Beilock et al., 2010; Cvencek et al., 2011), and dwindle their interest in pursuing STEM-related studies and professions (Schmader et al., 2004; Kiefer and Sekaquaptewa, 2007a; Watt et al., 2012; Wang and Degol, 2013; Ertl et al., 2017).

Cognitive performance may also be affected by mere awareness of, rather than belief in, stereotypes of the different abilities of targeted groups of persons. Thus when situational variables implicitly or explicitly activate stereotypes, they might induce a so-called ‘stereotype threat’ in the negatively stereotyped group members, and promote a reduction in their confidence and cognitive performance in those tasks perceived as being relevant to the activated stereotype (Steele and Aronson, 1995; Maass and Cadinu, 2003; Pennington et al., 2016). Accordingly, several studies have shown that the situational cues (e.g., received task instructions) that explicitly state or implicitly activate gender-related stereotypes reduce females’ performance in experimental tasks and tests measuring visuospatial abilities (McGlone and Aronson, 2006; Moè and Pazzaglia, 2006; Campbell and Collaer, 2009; Hausmann et al., 2009; Heil et al., 2012; Neuburger et al., 2015). However by encouraging downward social comparisons with a denigrated outgroup, the same situational conditions to promote stereotype reactivation might boost self-confidence and performance in non-negatively stereotyped groups (Blanton et al., 1999; Walton and Cohen, 2003). Accordingly, the explicit or implicit activation of stereotypes on the allegedly different visuospatial abilities of males and females also results in increased male performance in MRT (Moè and Pazzaglia, 2006; Campbell and Collaer, 2009; Hausmann et al., 2009), and in other cognitive domains ordinarily perceived as “masculine” (e.g., math; Kiefer and Sekaquaptewa, 2007b).

Although these and other studies clearly establish that endorsement, implicit interiorization or situational activation of gender-related stereotypes might promote opposite effects in males and females’ performance in math and visuospatial tasks, less is known about the individual variables that can moderate these effects (Maass and Cadinu, 2003). This is partly due to the generalized experimental treatment of females and males as being two distinct, but internally, homogenous groups and is also owing to focalization on average-based comparisons. Therefore, in the present study, we decided to compare subgroups of females and males with presumably different degrees of visuospatial abilities (STEM-Males ≥ STEM-Females > HUM-Males ≥ HUM-Females) and stereotypic gender-science beliefs/associations (STEM-Males = HUM-Females > HUM-Males > STEM-Females; see Nosek and Smyth, 2011) in a mixed design that allowed us to establish statistical relationships within, between and across groups.

More specifically, we assessed the relationship between the implicit and explicit gender-science biases held by a single cohort of female and male students of STEM and humanities’ majors and their MRT performance after receiving stereotype-congruent (“males will do better”), stereotype-incongruent (“females will do better”) or stereotype-nullifying (“no gender differences are expected”) experimental instructions. After taking into account the results of previous studies, we hypothesized that 3DMRT performance should relate to the interactive effects between the academic trajectory (STEM *vs.* humanities) and situational variables (received instructions) rather than their raw categorization as females or males. In this way, by reactivating preexisting gender-related explicit beliefs/implicit associations, the received instructions should differentially modify 3DMRT performance in each group and promote specific constellations of between-group differences in each experimental condition. These differences were expected to be larger after receiving stereotype-congruent instructions, when task difficulty increased and among participants endorsing stereotypic views of females and males (a more specific hypotheses’ formulation is provided in the different subheadings of the Results section). Moreover, correlational and linear-regression analyses were used to specifically explore whether the influence of gender-science biases on the participants’ 3DMRT performance was: (1) similar in females and males; (2) similar in STEM and humanities students; (3) similar across the different experimental conditions. Finally, mediation analyses were used to test the *a priori* hypothesis that these gender-related biases influence 3DMRT performance by decreasing/ increasing the participants’ self-confidence.

## Materials and Methods

This study was carried out in accordance with the recommendations of the ethical standards of the American Psychological Association. The protocol was approved by the Ethics Standards Committees of the Universitat Jaume I. In accordance with the Declaration of Helsinki all subjects gave written informed consent prior to participating.

### Participants

Participants were university students at the Universitat Jaume I (Spain) who self-volunteered in response to an invitational email. To be included in the study, participants had to meet the following inclusion criteria: (1) to be in their first university year; (2) to maintain a consistent academic specialization in STEM or humanities since the last two high school years. The initial sample comprised 110 subjects, but five subjects were excluded from the statistical analysis due to incomplete reports of relevant demographic data or to violations of the inclusion criteria. Thus, 105 participants were included in this study (see **Table 1** for the sample details), which were subdivided into four groups according to their self-reported gender and college major. Two of these groups, STEM males (STEM-M; *N* = 30) and Humanities females (HUM-F; *N* = 25), had stereotypic gender-major combinations and the other two, STEM females (STEM-F; *N* = 28) and Humanities males (HUM-M; *N* = 22), had non-stereotypic gender-major combinations. All the participants signed informed consent and their collaboration was awarded with €20.

**Table 1:** The sample’s demographic and academic characteristics.

	Males	Females
Computer sciences	10	8
Engineering	20	20
Total STEM	30	28
Journalism	8	8
Education	5	15
Other humanities studies	9	2
Total HUMANITIES	22	25
Total participants	52	53
Age	19.10 ± 1.20	18.96 ± 1.30

*This table presents the number of participants of each gender and major. Male and female participants’ ages (mean ± SD) are in the bottom row.*

### Measures

All the experimental tasks were programmed and presented in individual personal computers using the Millisecond Inquisit software package 4.0 (Millisecond©). The experimental tasks completed by all the participants included in presentation order: a demographic data form (on which participants reported their gender, age and university major), a mental rotation task, the Gender-Science implicit association test (IAT) and a single-item question to assess explicit beliefs on the suitability of females and males for scientific studies/professions.

#### 3D Mental Rotation Task (3DMRT)

To construct our 3DMRT task, we used the stimuli set developed and validated by Ganis and Kievit (2015). As in the classical paper-and-pencil mental rotation task designed by Shepard and Metzler (1971), each stimulus displays two abstract figures (a baseline object and a target object) composed of 7–11 cubes, arranged on four arms and connected end-to-end in a sequence. Ganis and Kievit (2015) provided eight different stimuli variations, grouped into two main categories: four “same” stimuli (those at which the baseline and target objects can be made to coincide with each other through a 0°, 50°, 100°, or 150° rotation on the vertical axis) and four “different” stimuli [whenever this is not possible, one figure arm (or more) is flipped]. Thus, by using the different rotation angles of a single figure, this set of stimuli allows the parametric manipulation of task difficulty. Furthermore, since the number of cubes and other characteristics of figures are identical in “different stimuli” and “same stimuli,” the task cannot be carried out merely by taking into account the number of cubes in the objects or any other spurious cue.

Our 3DMRT comprised three phases, which correspond to three experimental conditions, each preceded by a different set of instructions (see Procedure). In all these experimental phases, we used six versions (2 categories × 3 rotation angles, 50°, 100°, and 150°) of eight different stimuli across 48 time-restricted trials (duration: 7.5 s; ITI: 0.5 s). These time parameters were the same as those used by Ganis and Kievit (2015) when validating the current stimuli set. Their inclusion was a necessary control to ensure a similar task performance pace for all the participants, which allows administering the necessary instructions before each experimental phase. In each trial, the computer screens displayed a baseline (left) and a target figure (right). The target figure could be a “same” or a “different” rotated (50, 100, or 150°) version of the baseline figure, but both figures had the same number of cubes and arms arrangement in all cases. The participants were asked to respond by pressing the “b” key (masked with a green tag) on their computer keyboard if they decided that the objects in a pair were the same, or by pressing the “n” key (masked with a red tag) if they decided that the two objects differed. Accuracy (number of correct responses) and latency to respond were automatically measured and, at the end of each phase, subjects were asked to provide (by means of a sliding bar of 10 discrete steps) an estimation of the percentage of correct responses achieved. This additional requirement provided an overall measure confidence in task execution, similar to that used by Estes and Felker (2012).

#### Implicit Association Test

The Implicit Association Test (Greenwald et al., 1998) is commonly used to assess implicit stereotypic associations, such as those which differentially link males to sciences and females to humanities (Nosek et al., 2002; Smyth and Nosek, 2015). For this study, the Gender-Science IAT script provided at http://www.millisecond.com/download/library/ (the Milisecond Test Library) was adapted to and translated into Spanish for this study (see Supplementary Table 1). This provided script implements the standard IAT procedure, which consists of 7 phases.

##### Phase 1 (Target category sorting training; 20 trials)

Participants are asked to discriminate and classify the target stimuli (male/female names) that appear at the center on the screen into one of the two categories (female/male) displayed in top corners by pressing the left (“E”) or the right (”I”) key on the computer’s keyboard.

##### Phase 2 (Attribute sorting training; 20 trials)

Participants are asked to similarly classify attribute stimuli (majors) into one of the two categories (humanities/ STEM) displayed in the top corners of the computer’s screen using the same keys than in the previous phase.

##### Phase 3 (Test block. Stereotype consistent target-attribute pairing; 20 trials)

Participants are asked to perform a combined categorization task by responding with the “E” key to both target and attribute stimuli belonging to the categories (female/humanities) placed on the left top corner and with the “I” key to both target and attribute stimuli belonging to the categories (male/ STEM) displayed on the right top corner of the computer screen.

##### Phase 4 (Test block; Stereotype consistent target-attribute pairing)

This phase is identical to the previous one but consists of 40 trials.

##### Phase 5 (Target category sorting training; 20 trials)

This phase is identical to phase 1 but the target sides are switched, so participants must classify male names by pressing the “I” key and the female names by pressing the “E” key. Twenty trials.

##### Phase 6 (Test block. Stereotype inconsistent target-attribute pairing; 20 trials)

This phase is identical to phase 3, but the category-attribute pairs are reversed. Thus, female names and STEM majors share the same response key (“E”) whereas the male names and humanities majors are classified by pressing the “I” key.

##### Phase 7 (Test block. Stereotype inconsistent target-attribute pairing)

This phase is identical to the previous one but consists of 40 trials.

The provided script automatically counterbalances the order presentation of phases 3–4 and 5–6, so half of the participants perform first the test blocks containing stereotype consistent trials and the other half the stereotype inconsistent test blocks. This script also automatically calculates the so-called *d*-scores (Greenwald et al., 2003). *d*-scores are standardized deviation scores that range between +2 and -2, whose interpretation is similar to that of Cohen’s *d* statistics. Following the general convention, the IAT protocol used herein were arranged to provide positive *d* values for stereotype-consistent associations (e.g., “science = male/humanities = female”) and negative d values for stereotype-inconsistent associations.

#### Explicit Beliefs

Participants were asked to explicitly declare and quantify their beliefs as to whether males and females differ in their suitability for “scientific tasks.” We literally posed this question as “Who is better suited for science?” and the participants provided answers by a sliding bar of 10 discrete steps. Thus, setting the bar at 1 and 10 indicated that males/females were maximally suited for science, respectively (while setting it at 5 indicated no differences in this respect). Individual scores were computed as 5, minus the provided answer. In this way, and similarly to the IAT *d*-scores, positive (1–4) values quantified the presumed differences to favor males and negative (-1 to -4) values quantified the presumed differences to favor females.

### Procedure

The experiment was carried out during six different experimental sessions, and each session involved 15–20 participants. As group composition might create a threatening environment for negatively stereotyped groups (Inzlicht and Ben-Zeev, 2000), we matched the participants in each session for gender and academic specialization into four similarly sized groups (see Supplementary Table 2). At the beginning of each session, three female experimenters greeted the participants in the laboratory, and they randomly assigned them to an individual desk equipped with a personal computer. After giving their informed consent, the experimenters asked the participants to fill in the *Demographic data* form and to wait for further instructions.

After all the participants had completed this first step, a senior researcher introduced the 3DMRT task, and informed them that it comprised three successive phases that should be initiated after her explicit instructions. Before starting each phase, and with the help of a video projection system, the researcher explained the task generalities (goal, response keys, etc.) and provided the specific instructions for each experimental condition. Phases were labeled and presented to the participants as “optimized for women,” “optimized for men” and “neutral.” The experimenter also emphasized that the selection of the stimuli of each phase was in accordance with previous studies in which they proved to be differently processed and resulted in enhanced performance for females or males, or had led to similar results between genders, respectively. These explanations came along with faked figures of brain scans and bar graphs, which displayed such differential results, which also appeared in the written instructions that the participants had to individually read on their computer screens before starting each phase. In order to increase distinguishability between conditions, the stimuli of the “optimized for women,” “optimized for men” and “neutral” conditions appeared on a pink, a blue and a white background, respectively (see Supplementary Figure 1). The order of these three experimental conditions was randomized across the six experimental sessions as a strategy to prevent any practice/learning effect (see Supplementary Table 3).

After finishing the 3DMRT task, the same leading researcher introduced the IAT as a word-sorting task by carefully avoiding any reference to gender or gender-related differences and provided the pertinent instructions for its completion. This cautious introduction to the IAT intended to minimize the chance of any carry-over effects from previous experimental phases. The provided instructions, which emphasized responding quickly, but accurately, also came in writing, shown on the individual screen of each participant’s computer before starting the IAT.

Finally, participants were instructed to answer a single explicit question to assess their beliefs as to whether males or females are more capacitated for science (see *Explicit Beliefs* in the Measures section). Once they answered this question, participants were thanked and economically rewarded for their participation.

### Data and Statistical Analyses

All the data included in the present study are provided as Supplementary Material (Supplementary Data Sheet S1). Data were analyzed using SPSS 23 (IBM Corp.) and PRISM 7.0 (GraphPad Inc.) for Mac OS X. Figures were constructed using PRISM 7.0 GraphPad Inc.).

One-sample Student’s *t*-tests were used to evaluate whether or not the explicit beliefs and implicit associations held by each group of participants were significantly different from zero. Between-group differences in these variables as well as in the observed and expected 3DMRT performance were evaluated by design-appropriate ANOVAs, followed by Tuckey HSD *post hoc* comparisons. The relationship between explicit and implicit gender-related biases and observed/expected 3DMRT performance was initially evaluated by means of Pearson’s *r* correlation index. However, in a second step, linear-regression-based procedures were used to explore in further detail the relationship between the IAT scores and observed/ expected 3DMRT performance scores. These more fine-grained analyses included: (1) the evaluation of a possible moderating effect of academic specialization on the influence of implicit gender-science associations over the observed and expected 3DMRT performance scores; (2) The evaluation of a possible mediatory role of confidence on the effects of these implicit associations on the observed 3DMRT performance.

## Results

### Explicit Beliefs and Implicit Associations

H1: The participants, especially those of groups with gender-major stereotypic combinations, will hold explicit beliefs and implicit associations that preferentially link science to males and humanities to females.

**FIGURE 1:**
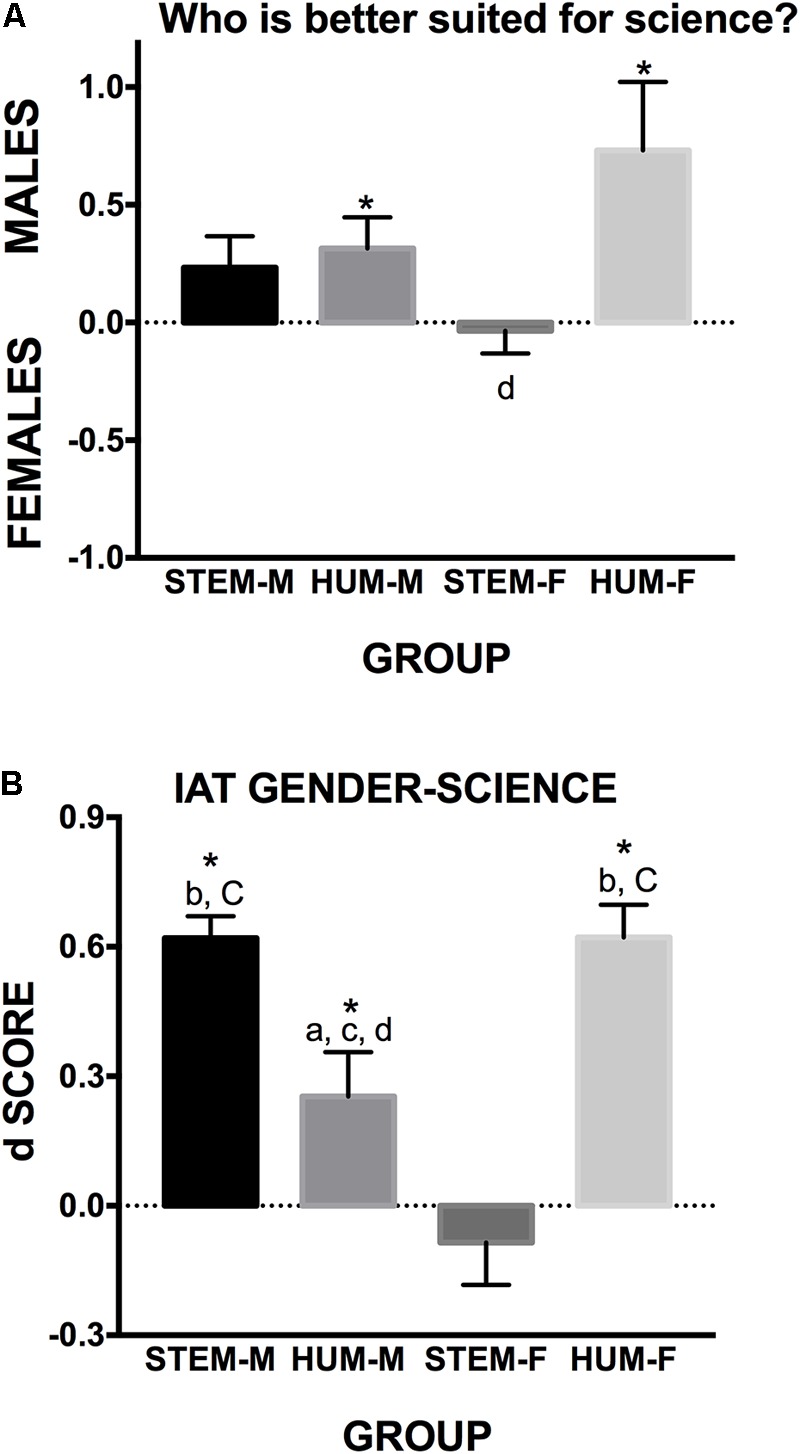
Explicit beliefs and implicit associations. **(A)** Depicts explicit beliefs as to a different suitability of males and females for science studies/professions. **(B)** Illustrates the *d*-scores for IAT Gender-Science. ^∗^Significantly different from zero, *p* < 0.05; letters denote statistically significant differences between groups: A different from STEM-Males, B different from HUM-Males, C different from STEM-Females and D different from HUM-Females (capital letters, *p* < 0.01; lowercase letters, *p* < 0.05).

To ascertain whether or not the participants held explicit beliefs as to a differential suitability of females and males for science, one-sample Student’s *t*-tests were used. As shown in **Figure 1A**, the size of this belief significantly differed from zero in HUM-Males (*t*_21_ = 2.309, *p* < 0.05) and HUM-Females (*t*_24_= 2.520, *p* < 0.05), and approached statistical significance in the STEM-Males group (*t*_29_= 1.756, *p* = 0.09). In a second step, we analyzed the between-group differences by means of one-way ANOVA. The group factor reached statistical significance (*F*_3,101_= 2.86; *p* < 0.05; $\eta_{p}^{2}$ = 0.091) which, as revealed by the Tukey HSD *post hoc* comparisons, was driven solely by a difference between the HUM-Females and the STEM-Females groups (*p* < 0.05, Cohen’s *d* = 0.739).

**FIGURE 2:**
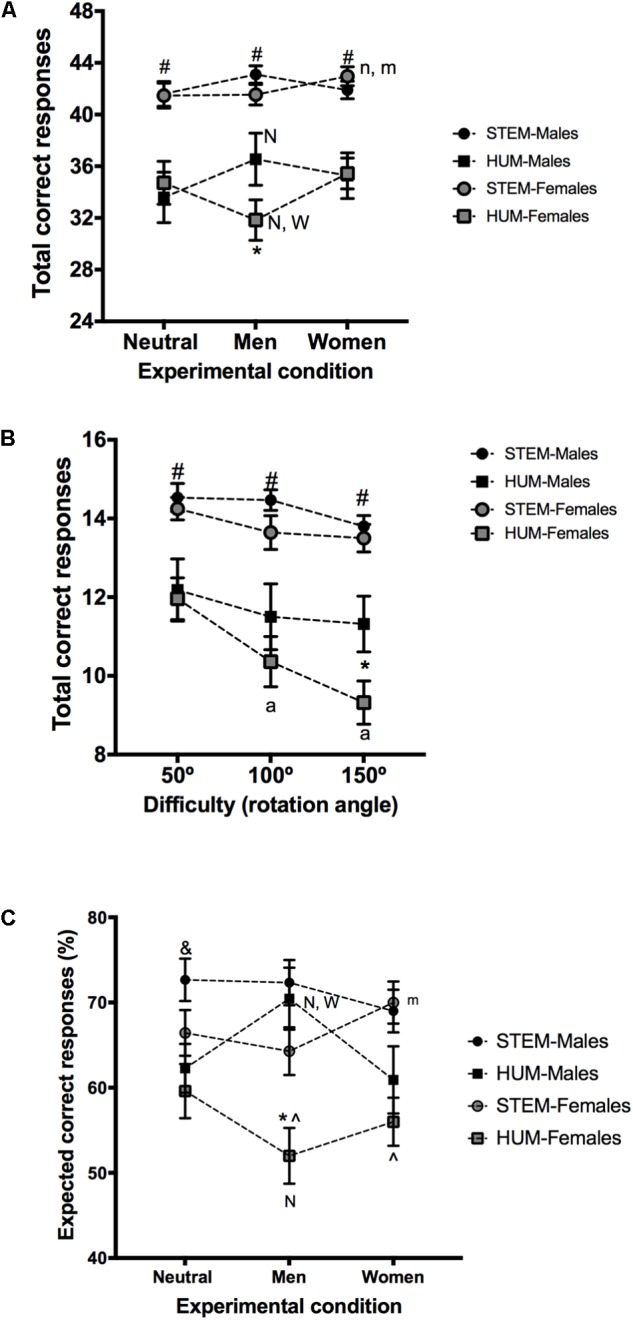
3DMRT observed and expected performance. **(A)** Depicts the observed performance (mean ± SEM of the number of correct responses) in a mental rotation task run under three experimental conditions (“neutral,” “optimized for men” and “optimized for women”). **(B)** Illustrates the relationship between task difficulty (rotation angle) and 3DMRT observed performance (mean ± SEM of correct responses) for each participant’s group for the “optimized for men” condition. **(C)** Shows expected performance (mean ± SEM of the participants’ expected percentage of correct responses) as a measure of task execution confidence in each experimental phase (see the Measures section for details). Note that in **(A,B)**, the *Y*-axis were adjusted to denote optimal and chance levels performance [^#^Significantly different from HUM groups; ^∧^Significantly different from STEM groups; ^∗^Significantly different from HUM-Males; ^&^Significantly different from HUM-Females; N, n Significantly different from the “neutral” condition (*p* < 0.01 and *p* < 0.05, respectively); W Significantly different from the “optimized for women condition” (*p* < 0.01); m Significantly different from the “optimized for men” condition (*p* < 0.05); a Significantly different from the 50^o^ rotation angle (*p* < 0.05)].

On the other hand, one-sample Student’s *t*-test revealed that STEM-Males (*t*_21_= 12.29, *p* < 0.001), HUM-Males (*t*_21_= 2.46, *p* < 0.05) and HUM-Females (*t*_24_= 8.24, *p* < 0.001), but not STEM-Females (*t*_27_= -0.872, *p* = 0.391), exhibited a significant implicit “male-science/female-humanities” stereotypic association (**Figure 2B**). A one-way ANOVA (*F*_3,101_= 18.12, *p* < 0.001; $\eta_{p}^{2}$ = 0.350) yielded a group effect on the size of this bias. The Tukey HSD *post hoc* comparisons revealed that this bias was larger in groups with gender-major stereotypic combinations than in those with non-stereotypic combinations (STEM-Males > HUM-Males: *p* < 0.05, Cohen’s *d* = 1.01; STEM-Males > STEM-Females: *p* < 0.01, Cohen’s *d* = 1.74; HUM-Females > HUM-Males: *p* < 0.05; Cohen’s *d* = 0.89; HUM-Females > STEM-Females: *p* < 0.01; Cohen’s *d* = 1.57). This bias was also larger in HUM-males than in STEM-Females (*p* < 0.05; Cohen’s *d* = 0.65).

These results confirmed Hypothesis 1. However, explicit beliefs and implicit associations are two distinct cognitive and poorly correlated (*r* = 0.147, *p* = 0.134) constructs and, only in the second one, the groups with gender-major stereotypic combinations (STEM-Males and HUM-Females) clearly obtained higher bias scores than those with non-stereotypic combinations (STEM-Females and HUM-Males).

### 3DMRT Observed Performance

H2: The experimental groups will differ in their observed 3DMRT performance (STEM-Males ≥ STEM-Females > HUM-Males ≥ HUM-Females).

H3: The received instructions will differentially modify 3DMRT performance in each group and will hence lead to specific constellations of between-group differences in each experimental phase.

H4: The ability of the experimental instructions to promote gender-related differences in 3DMRT performance will increase with task difficulty.

3DMRT performance was assessed by two main variables: latency to respond and the number of correct responses. As latencies to respond did not differ between groups for any experimental condition (Supplementary Table 4), we do not discuss them further.

Regarding the number of correct responses (**Figure 2A**), a two-way repeated measures ANOVA revealed a significant effect for the group factor (*F*_3,101_ = 17.16, *p* < 0.001; $\eta_{p}^{2}$ = 0.338), but not for the condition factor (*F*_2,202_= 3.08, *p* = 0.18), although the interaction between both factors was significant (*F*_6,202_= 2.98, *p* < 0.001; $\eta_{p}^{2}$ = 0.160). This significant group × condition interaction allowed us to explore how the performance of each group varied across the three experimental conditions (within group comparisons) as well as the between group differences for each one.

#### Effects of the Received Instructions in Each Experimental Phase (Within Group Comparisons)

The Tukey HSD-based comparisons showed that the performance of the two STEM groups remained largely stable across the three experimental conditions. However, the less conservative Student’s *t*-tests for related samples revealed a slight enhancement of STEM-Females’ performance for the “optimized for women” condition compared to the other two (“neutral”: *t*_27_= 2.075, *p* = 0.048; Cohen’s *d* = 0.332; “optimized for males”: *t*_27_= 2.655, *p* = 0.013; Cohen’s *d* = 0.352). The same *t*-test based analysis did not reveal any significant variation in the STEM-Males group.

The experimental phase had more pronounced effects on the HUM groups. The intra-group Tukey HSD-based comparisons revealed that HUM-Females’ performance dropped for the “optimized for men” condition to become lower than under the “neutral” (*p* < 0.05, Cohen’s *d* = -0.364) and the “optimized for women” (*p* < 0.05 Cohen’s *d* = -0.528) conditions. Conversely, HUM-Males displayed increased performance under the “optimized for men” condition, which became significantly higher (*p* < 0.05, Cohen’s *d* = 0.318) than for the “neutral” condition.

#### Between-Group Differences in Each Experimental Phase

Under all the experimental conditions STEM-Females and STEM-Males outperformed HUM-Females and HUM-Males (Tukey HSD *p* < 0.01; Cohen’s *d*, ranging from 0.98 to 1.83), but no differences between the two STEM groups were observed. HUM-Males outperformed HUM-Females for the “optimized for men” condition (*p* < 0.05, Cohen’s *d* = 0.557), but not for any other experimental condition.

Taken together, these results confirmed Hypotheses 2 and 3 by showing that the 3DMRT performance of STEM-Males, STEM-Females, HUM-Males and HUM-Females differed, and that some of their differences (remarkably those between genders) only arose when receiving gender-loaded task instructions.

#### Task Difficulty and Gender-Related Differences in 3DMRT Observed Performance

**Figure 2B** depicts the relationship between task difficulty (rotation angle) and the observed 3DMRT performance for each participants group for the “optimized for men” condition (the only one at which we observed gender-related differences). A two-way repeated measures ANOVA yielded significant group (*F*_3,101_= 17.16, *p* < 0.001; $\eta_{p}^{2}$ = 0.338), rotation angle (*F*_2,202_= 14.90, *p* < 0.001; $\eta_{p}^{2}$ = 0.128) and interaction (*F*_6,202_= 2.29, *p* < 0.05; $\eta_{p}^{2}$ = 0.064) effects.

All the groups showed rotation-related decreases in performance but, as revealed by the Tukey HSD *post hoc* comparisons, this effect was statistically significant only in HUM-Females (50° *vs.* 100° *p* < 0.01, Cohen’s *d* = 0.557; 50° vs. 150° *p* < 0.001, Cohen’s *d* = 0.977). The between-group comparisons revealed that the two STEM groups outperformed both HUMs groups, regardless of the rotation angle (*p* < 0.01 in all cases). Moreover, when difficulty was maximal (150°) HUM-Females gave fewer correct responses than HUM-Males (*p* < 0.05, Cohen’s *d* = 0.656), which hence confirms Hypothesis 4.

### Participants’ Expected 3DMRT Performance

H5: The received instructions will differentially modify the self-reported expected performance (confidence) in each group, which will then result in specific patterns of between-group differences in each experimental phase.

**Figure 2C** depicts the participants’ expected percentage of correct responses for each experimental condition. A two-way repeated measures ANOVA yielded significant effects for the group factor (*F*_3,101_= 6.94, *p* < 0.001; $\eta_{p}^{2}$ = 0.171) and for the group × experimental condition interaction (*F*_6,202_= 5.36, *p* < 0.01; $\eta_{p}^{2}$ = 0.137). This significant group x condition interaction allowed us to explore how this self-reported index of the participants confidence varied within each group across the three experimental conditions as well as the between-group differences in this variable under each experimental condition.

#### Effects of the Received Instructions in Participants’ Expected Performance in Each Experimental Phase (Within-Group Comparisons)

STEM males showed stable levels of expected performance across all the experimental phases. Conversely, all the other groups exhibited significant variations of expected performance depending on the received instructions. Thus HUM-Females’ expected performance dropped under the “optimized for men condition” and hence became significantly lower than for the “neutral” condition; Tukey HSD *p* < 0.05, Cohen’s *d* = 0.471). The opposite effect appeared for HUM-Males, with enhanced expected performance under the “optimized for men” condition (Tukey HSD *p* < 0.01, Cohen’s *d* = 0.566 and Tukey HSD *p* < 0.001, Cohen’s *d* = 0.657 compared to the “neutral” and the “optimized for women” conditions, respectively). Finally, the Student’s *t*-tests for related samples, but not the Tukey HSD-based comparisons, revealed a selective increase in STEM-Females’ expected performance under the “optimized for women” condition (*t*_27_= 2.741, *p* = 0.01; Cohen’s *d* = 0.261 and *t*_27_= 1.780, *p* = 0.08 compared to the “optimized for men” and “neutral” condition, respectively).

#### Between-Group Differences in Each Experimental Phase

The Tukey HSD *post hoc* comparisons revealed that HUM-Females had the lowest expected performance (confidence) scores in all the experimental phases. Thus, for the “neutral” condition, only the HUM-Females and the STEM-Males groups significantly differed (*p* < 0.01, Cohen’s *d* = 0.881). For the “optimized for women condition,” HUM-Females reported lower expected performance scores than STEM-Males (*p* < 0.01, Cohen’s *d* = 0.932), and also than STEM-Females (*p* < 0.01, Cohen’s *d* = 1.028). Finally, under the “optimized for men” condition, the HUM-Females group differed from all the other groups: STEM-Males (*p* < 0.001, Cohen’s *d* = 1.314), STEM-Females (*p* < 0.02, Cohen’s *d* = 0.789) and HUM-Males (*p* < 0.001, Cohen’s *d* = 1.138).

Taken together, the results of Sections “Effects of the Received Instructions in Participants’ Expected Performance in Each Experimental Phase (Within-Group Comparisons)” and “Between-Group Differences in Each Experimental Phase” confirmed hypothesis 5.

### Relationships Between Variables

H6: Observed and expected 3DMRT performance will be directly related between them, and will also show gender-dependent correlations with explicit beliefs and implicit associations preferentially linking males and science.

Observed and expected 3DMRT performance directly correlated with one another: (“neutral” condition *r* = 0.536, *p* < 0.000; “optimized for the men” condition *r* = 0.596, *p* < 0.000; “optimized for the women” condition *r* = 0.468, *p* < 0.000). Moreover, these performance-related variables correlated in a gender-dependent manner with the explicit and, more notably, the implicit “gender-science” biases (**Table 2**).

**Table 2:** Correlations by gender.

	Explicit		Implicit	
	Females	Males	Females	Males
Correct responses “neutral” condition	***r* = -0.277**	*r* = 0.053	***r* = -0.299**	***r* = 0.330**
	***p* = 0.044**	*p* = 0.710	***p* = 0.030**	***p* = 0.017**
Correct responses “optimized for men” condition	***r* = -0.366**	*r* = -0.030	***r* = -0.433**	***r* = 0.304**
	***p* = 0.007**	*p* = 0.834	***p* = 0.001**	***p* = 0.029**
50°	***r* = -0.311**	*r* = -0.042	***r* = -0.352**	*r* = 0.260
	***p* = 0.023**	*p* = 0.765	***p* = 0.010**	*p* = 0.060
100°	***r* = -0.280**	*r* = -0.146	***r* = -0.356**	*r* = 0.124
	***p* = 0.042**	*p* = 0.302	***p* = 0.009**	*p* = 0.383
150°	***r* = -0.327**	*r* = -0.215	**r = -0.452**	*r* = 0.239
	***p* = 0.017**	*p* = 0.125	***p* = 0.001**	*p* = 0.087
Correct responses “optimized for women” condition	***r* = -0.276**	*r* = -0.153	***r* = -0.470**	***r* = 0.345**
	***p* = 0.045**	*p* = 0.280	***p* < 0.000**	***p* = 0.012**
Expected correct responses “neutral” condition	*r* = -0.139	*r* = 0.005	*r* = -0.018	***r* = 0.303**
	*p* = 0.322	*p* = 0.996	*p* = 0.898	***p* = 0.029**
Expected correct responses “optimized for men” condition	*r* = -0.253	*r* = -0.022	*r* = -0.260	*r* = 0.199
	*p* = 0.067	*p* = 0.878	*p* = 0.060	*p* = 0.157
Expected correct responses “optimized for women” condition	***r* = -0.318**	*r* = -0.014	***r* = -0.310**	***r* = 0.337**
	***p* = 0.020**	*p* = 0.920	***p* = 0.024**	***p* = 0.014**

*Pearson’s *r* index was used to quantify the correlation between Gender-Science explicit beliefs and implicit associations (IAT *d* scores) and the different indexes of 3DMRT performance. Statistically significant correlations (*p* < 0.05) are highlighted in bold.*

The **Table 2** results confirm Hypothesis 6 and also show that the implicit “male-science/female humanities” associations are more closely related to 3DMRT performance than explicit beliefs. These results also indicate that the same “male-science/female-humanities” association might have opposite functional consequences on female and male 3DMRT performance.

In order to confirm this last observation, we calculated an IAT-derived “influence” index. More specifically, females IAT scores were multiplied by -1, and those of males by 1. This transformation does not change the strength of the implicit Gender-Science associations revealed by the IAT, but slightly modifies the interpretation of the performed correlations, which now provide an index of the expectable “influence” of these implicit gender-related associations on 3DMRT performance rather than a plain measure of their co-variation. As expected, this IAT-derived “influence” index correlated directly with the observed 3DMRT performance (“neutral” condition: *r* = 0.246, *p* < 0.02; “optimized for males” condition: *r* = 0.425, *p* < 0.000; “optimized for females” condition: *r* = 0.319, *p* < 0.001). Similar correlations were found for expected 3DMRT performance (“neutral” condition: *r* = 0.204, *p* < 0.04; “optimized for males” condition: *r* = 0.400, *p* < 0.000; “optimized for females” condition: *r* = 0.277, *p* < 0.005).

By means of this IAT-derived “influence” index, we sought to investigate three additional research questions:

(Q1) Does the implicit “male-science/female humanities” association equally affect 3DMRT observed and expected performance in STEM and humanities students?

**FIGURE 3:**
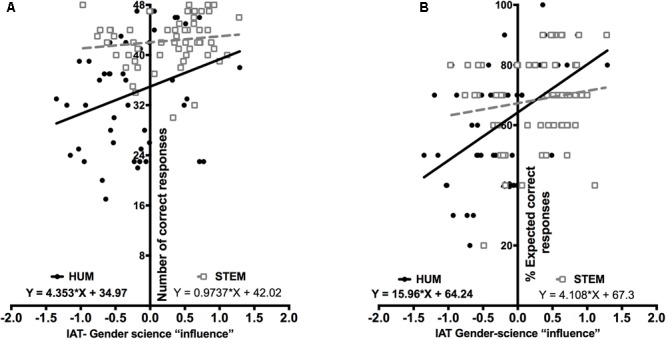
Academic specialization moderates the effects of implicit biases. The data depict individuals’ position in a bivariate space defined by the IAT Gender-Science “influence” (*X*-axis) and observed/expected 3DMRT performance (*Y*-axis). To assess the statistical moderation of academic specialization on the effects of implicit associations on MRT performance, separate linear regressions were calculated for HUM (black circles) and STEM (white squares) students. In these analyses, the IAT “influence” scores were used as regressors of the 3DMRT observed **(B)** and expected **(A)** performance scores. Only the slope of the regression equations of the HUM groups significantly differed from zero (in bold, *p* < 0.05 and *p* < 0.01 for observed and expected performance, respectively).

To answer this question, we used the regression-based moderation testing procedure proposed by Baron and Kenny (1986). Because the highest correlations between IAT-derived “influence” scores and performance measures were observed for the “optimized for men,” we focused on this condition. Regarding observed performance (**Figure 3A**), the slope of the regression line for the HUM group significantly differed from zero (*F*_1,45_= 4.47, *p* = 0.04), unlike that calculated for the STEM group (*F*_1,56_= 1.01, *p* = 0.31). These slopes showed a clear trend toward being significantly different between them (*Z* = 1.48, *p* = 0.06). Similarly, as shown in **Figure 3B**, the slope of the regression line for the expected performance of the HUM (*F*_1,45_= 16.25, *p* < 0.001), but not that of the STEM groups (*F*_1,56_= 1.24, *p* = 0.26), significantly differed from zero, and yielded a significant inter-groups difference in this case (*Z* = 2.19, *p* = 0.01). These results confirmed that academic specialization is a significant moderator of the IAT Gender-Science “influence” on the observed and expected 3DMRT performance, and revealed that this bias exclusively affected HUM students.

Confirming these results, we found significant correlations between the IAT “influence” scores and the observed and expected performance in HUM, but not in STEM, students (**Table 3**). The same correlational analysis revealed that the “influence” of the implicit “male-science/female-humanities” association on observed and expected 3DMRT performance varied for the different experimental conditions (see below).

**Table 3:** Correlations between the IAT “influence” scores and 3DMRT performance in HUM and STEM students.

	HUM	STEM
Correct responses “neutral” condition	*r* = -0.044	*r* = 0.171
	*p* = 0.771	*p* = 0.200
Correct responses “optimized for men” condition	***r* = 0.300**	*r* = 0.121
	***p* = 0.04**	*p* = 0.367
50^o^	*r* = 0.157	*r* = 0.123
	*p* = 0.802	*p* = 0.357
100^o^	*r* = 0.107	*r* = 0.123
	*p* = 0.473	*p* = 0.341
150^o^	***r* = 0.287**	*r* = 0.127
	***p* = 0.05**	*p* = 0.341
Correct responses “optimized for women” condition	*r* = 0.072	*r* = 0.123
	*p* = 0.630	*p* = 0.357
Expected correct responses “neutral” condition	*r* = 0.038	*r* = 0.005
	*p* = 0.802	*p* = 0.971
Expected correct responses “optimized for men” condition	***r* = 0.515**	*r* = 0.092
	***p* < 0.000**	*p* = 0.494
Expected correct responses “optimized for women” condition	*r* = 0.233	*r* = 0.056
	*p* = 0.114	*p* = 0.676

*Pearson’s *r* correlation indices and associated *p-*values are provided. Statistically significant correlations (*p* < 0.05) are in bold.*

(Q2) When do implicit biases affect expected and observed 3DMRT performance?

Several results of the present study were suggestive of a specific effect of the implicit “male-science/female humanities” association on the observed and expected 3DMRT performance of HUM, but not of STEM, students for the “optimized for men” condition. In order to confirm these effects and to explore their specificity, we ran a series of regression analyses.

As shown in **Table 4A**, the IAT “influence” scores (but not gender, age, university major, or explicit beliefs) achieved statistical significance as predictors of the observed 3DMRT performance of HUM students for the “optimized for men” condition. Similarly, the IAT “influence” was the only significant predictor of the expected performance of HUM students under this experimental condition (**Table 4B**). Conversely, neither the IAT “influence,” nor gender, age, university major or explicit beliefs achieved statistical significance as predictors of 3DMRT observed or expected performance of HUM students for the “neutral” or the “optimized for women” conditions, nor as predictors of STEM students’ performance. Therefore, a specific effect of the implicit “male-science/female humanities” association on HUM students’ 3DMRT performance was confirmed.

**Table 4:** Step-forward linear regression of the **(A)** observed and **(B)** expected performance of HUM students for the “optimized for men” condition.

		Beta	*t*	*p*-value
**(A) Observed performance**				
Included in the	Constant	–	26.45	<0.000
model	IAT “influence”	0.300	2.11	0.04
Excluded from	Age	0.194	1.33	0.18
the regression	Gender	–0.107	–0.51	0.60
model	University major	–0.182	–1.24	0.21
	Gender-science explicit belief	–0.232	1.65	0.10
	Model summary	*R*	Adjusted *R*^2^	*p*-value
		0.300	0.07	0.04
**(B) Expected performance**				
Included in the model	Constant	–	25.26	<0.000
	IAT “influence”	0.515	4.02	<0.000
Excluded from	Age	0.197	1.51	0.13
the regression	Gender	–0.27	–1.50	0.14
model	University major	–0.124	–1.24	0.35
	Gender-science explicit belief	–0.178	–1.39	0.16
	Model summary	*R*	Adjusted *R*^2^	*p*-value
		0.515	0.249	<0.000

*Separate stepwise forward linear regression analyses were conducted in the HUM and STEM groups to compare the predictive power of the IAT “influence” scores and of other possible predictors on the 3DMRT observed and expected performance at each experimental condition. For these analyses, nominal variables were coded as follows: gender (males = 1, females = 2) and university major (computer sciences = 1, engineering = 2, journalism = 3, education = 4, other humanities’ studies = 5). Similar regression analyses were conducted for STEM students, but no variable entered in the model).*

(Q3) Does expected performance (confidence) mediate the effects of implicit “male-science/female-humanities” association on 3DMRT observed performance?

Previous studies (Steele, 1997; Walton and Cohen, 2003; Estes and Felker, 2012) have suggested that, by reducing confidence, gender stereotypes promote decrease female performance in “male cognitive domains,” such as math or mental rotation. Therefore, we sought to explore whether our measure of confidence (expected performance) would mediate the “influence” of implicit gender-science associations on the 3DMRT observed performance. Taking into account all the previous results of our own study, we should solely observe this effect in HUM students (the only ones who displayed gender-related differences) and under the “optimized for men” condition (the only one at which we observed these differences). We tested this *a priori* hypothesis following the regression method for simple mediation described by Baron and Kenny (1986).

**FIGURE 4:**
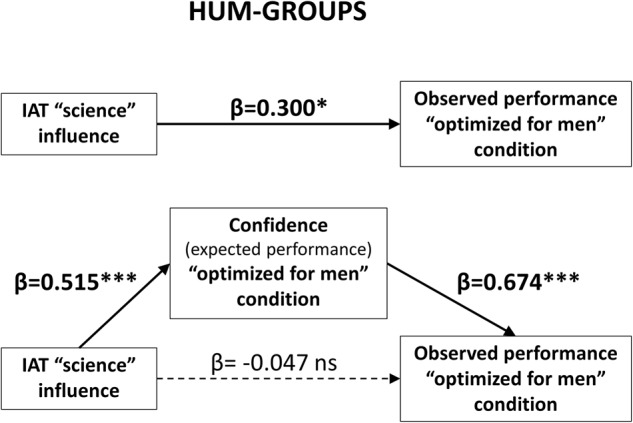
Expected performance (confidence) mediates the effects of implicit gender-science associations. Mediation analysis was performed according to the 3-steps regression method described by Baron and Kenny (1986). First, we confirmed that the IAT “influence” scores predicted 3DMRT observed performance (β = 0.300, *p* < 0.05). Then we confirmed that these scores also predicted confidence (expected performance; β = 0.515, *p* < 0.000) and that confidence predicted observed performance (β = 0.650, *p* < 0.000). Finally, we simultaneously included the IAT “influence” and confidence scores as predictor variables of 3DMRT observed performance in a single regression equation. In this crucial step, only the confidence (expected performance) scores remained as strong predictors (β = 0.674, *p* < 0.000) of observed performance, while the predictive value of IAT “influence” scores became almost zero (β = –0.047, *p* = 0.727), hence revealing a near complete mediatory effect of confidence (^∗^*p* < 0.05, ^∗∗∗^*p* < 0.000; ns, non-significant).

As shown in **Figure 4**, when the IAT “influence” and expected performance scores were simultaneously included in a single regression equation, only the second remained a strong predictor (β = 0.674, *p* < 0.000) of observed performance, while the predictive value of IAT “influence” scores’ came very close to zero (β = -0.047, *p* = 0.727. That is, when the effect of confidence was taken into account, the influence of the implicit “male-science/female-humanities” association in HUM students’ 3DMRT performance was entirely eliminated. The specificity of this mediatory effect was ratified by testing several alternative models with the same regression-based procedure. These additional tests included assessing: (1) the same model in STEM students under the “optimized for men” condition; (2) the same model in HUM students under the “neutral” and “optimized for women” conditions; (3) the reverse model (observed performance mediates expected performance of HUM students under the “optimized for men” condition). As expected, the results of all these tests were negative (for details, see the figures and text included in the Supplementary Materials Image 1 file).

## Discussion

Our main results can be summarized as follows: (1) university students hold explicit beliefs and implicit associations that preferentially link science to males and humanities to females; (2) participants’ science-related beliefs and associations vary according to an academic specialization (STEM *vs.* humanities) *per* gender interaction; (3) under experimental conditions specifically aimed to nullify or counteract these participants’ stereotypic beliefs and associations, academic specialization was the only relevant predictor of 3DMRT performance; (4) when the received experimental instructions reactivated participants’ stereotypes on gender-visuospatial abilities, explicit beliefs and, more significantly, gender-science implicit associations, were able to affect 3DMRT performance; (5) changes in confidence mediated these effects and academic specialization moderated them.

### Explicit and Implicit Gender-Science Biases

The stereotypical notion of males being more suited for science was explicitly endorsed by the HUM-Males and, to a larger extent, by the HUM-Females groups (**Figure 1A**). As expected from previous studies (Greenwald and Banaji, 1995; Nosek et al., 2002), this explicit belief did not significantly correlate with the implicit Gender-Science associations revealed by the IAT and correlated solely with 3DMRT performance in females, but not in males (**Table 2**). This observation, together with the results of our linear regression-based analyses (see **Table 4**, but also Supplementary Tables 5–7) and those of some previous studies (Hyde et al., 1990; Schmader et al., 2004; Nosek et al., 2009), suggest that explicit gender-science beliefs are less accurate predictors and/or less powerful influencers of cognitive performance than implicit attitudes.

The participants also exhibited an implicit “science-male/humanities-female” association that correlated significantly with the 3DMRT performance in both females and males (**Table 2**). This bias was larger among the gender-major stereotypic combination groups (STEM-Males = HUM-Females; **Figure 1B**) than in those with non-stereotypic combinations (HUM-Males > STEM-Females). This observation is in agreement with cognitive-consistency principles (Nosek et al., 2002), with the results of a massive online survey conducted with college-educated people (Smyth and Nosek, 2015), and also with studies which show that STEM-majoring females hold weaker implicit gender-math stereotypes than both males from the same field and female and male humanities students (Nosek and Smyth, 2011; Smeding, 2012). Taken together, these studies suggest that the implicit “science- male/humanities-female” association is highly related to academic/ professional career orientation. Moreover, since our study was conducted in freshman students, our results show that this implicit association is acquired before starting university and suggest that it might influence the students’ choice of college major, hence contributing to the asymmetrical representation of girls and boys in STEM and humanities studies.

### Interaction Between Implicit Associations and “Neutralizing,” “Stereotypic” and “Counter-Stereotypic” Instructions and Its Effects on 3DMRT Performance

When interacting with situational cues (received instructions), the implicit “male-science/female-humanities” association was able to influence 3DMRT performance. As expected, the effects of this implicit bias were substantially smaller when arranging situational cues to nullify latent stereotypes (“neutral” condition) than under the experimental conditions which aimed to activate them (see the correlation values in **Tables 2, 3**). Indeed, under this “stereotypes’ neutralizing condition,” STEM-students outperformed HUM-students, and no gender-related differences between these high and low performance groups were found (**Figure 2A**). Accordingly, regression analyses revealed that neither gender nor gender-related explicit beliefs or implicit associations were relevant predictors of 3DMRT performance under this experimental condition, which was significantly related only to academic specialization (see Supplementary Table 5). Thus our results confirm those of previous studies (Quinn and Spencer, 2001; Campbell and Collaer, 2009; Marchand and Taasoobshirazi, 2013), which also observed that stereotype nullification by experimenter-controlled cues suppressed gender-related differences in visuospatial abilities and other cognitive domains for which males’ superiority has been traditionally reported. As discussed below, these observations have important theoretical implications in the study and interpretation of “sex-differences” but also practical implications when trying to design educational interventions aimed to increase the representation of girls and women in STEM majors and professions.

The introduction of counter-stereotypic gender-related instructions (“optimized for women condition”) did not substantially change the groups’ 3DMRT performance. In this atypical situation, STEM-students displayed higher task accuracy than HUM-students but, once again, no gender-related differences were found (**Figure 2A**). Accordingly, linear regression-based analyses revealed that academic specialization, but not participants’ gender, gender-related beliefs or implicit associations, became a significant predictor of 3DMRT performance under this experimental condition (see Supplementary Table 7). However, the “optimized for women” and “neutral” conditions were not identical as only the former promoted a slight enhancement of observed and expected performance in STEM-, but not HUM-, females (**Figures 2A,C**). The different reaction of STEM- and HUM-Females to counter-stereotypic instructions could lie in their distinct *a priori* beliefs and implicit associations (**Figure 1**). Thus, lacking any explicit or implicit Gender-Science bias, STEM-Females benefited from females’ encouraging instructions, whereas the high and self-demoting biases held by HUM-Females made it impossible for them to benefit from the same positive endorsement. These observations replicate those made in previous studies (Moè and Pazzaglia, 2006; Wraga et al., 2006; Moè, 2009; Heil et al., 2012), which also found that instructions which stressed females’ superiority in mental rotation tasks increased their performance, and that this increase was more marked for those females who did not sustain *a priori* beliefs about males’ visuospatial superiority (Moè and Pazzaglia, 2006). However, in line with some (Moè, 2009; Heil et al., 2012), but not with other (Moè and Pazzaglia, 2006; Wraga et al., 2006) preceding studies, counter-stereotypic instructions did not bring about any change in STEM- or HUM-Males task performance. The reasons why these studies found distinct results remain unclear, but they might be indicative of a relatively weaker capacity of counter-stereotypic instructions to induce 3DMRT performance changes, especially if they result in a threat, and/or if subjects subscribe to the stereotypes contradicted by received instructions.

In contrast, stereotype-congruent instructions resulted in significant gender-related changes in 3DMRT performance. More specifically under the “optimized for men” condition, the 3DMRT accuracy of HUM-Females markedly diminished, but substantially increased in HUM-Males, and hence became significantly different between them and from their own performance under the other two experimental conditions (**Figure 2A**). Thus our results agree with those of previous studies, which have shown that experimental instructions which explicitly state females’ inferiority in visuospatial abilities reduce females’ performance in mental rotation tasks (Martens et al., 2006; Moè and Pazzaglia, 2006; Wraga et al., 2006; Campbell and Collaer, 2009; Hausmann et al., 2009; Moè, 2009; Heil et al., 2012), but increases males’ performance (Moè and Pazzaglia, 2006; Campbell and Collaer, 2009; Hausmann et al., 2009).

In line with this, it has been proposed that confidence might underlie between gender differences in 3DMRT performance (Estes and Felker, 2012) as well as instructions-driven performance changes in gender-stereotyped cognitive domains (Steele, 1997; Walton and Cohen, 2003). More specifically, it has been suggested that stereotype reactivation might induce a self-confidence threat that disrupts task performance in the negatively stereotyped group (Schmader et al., 2008), but may induce a self-confidence boost that increases performance in the non-negatively stereotyped group (Blanton et al., 1999; Walton and Cohen, 2003). In agreement with this proposal, we observed that (probably by re-activating previously held stereotypic associations; **Figure 1B** and **Table 3**) the stereotype-congruent instructions of the “optimized for men” condition promoted disparate changes not only in the 3DMRT performance of the HUM-Females and HUM-Males groups (**Figure 2A**), but also in their confidence (**Figure 2C**), and that confidence mediates the influence of implicit associations on 3DMRT observed performance (**Figure 4**).

However, stereotype-congruent instructions do not uniformly affect females or males’ performance as academic specialization moderates their effects (**Figure 3**). Accordingly, gender as a binary category did not come over as a significant predictor of 3DMRT performance for the “optimized for men” condition, which was instead mainly predicted from participants’ academic specialization (Supplementary Table 6). Moreover, although the IAT “influence” scores were also significant predictors of 3DMRT performance under this experimental condition (Supplementary Table 6), their effects were restricted to HUM students (**Table 4**). Thus, despite having very different implicit Gender-Science associations (**Figure 1B**), the 3DMRT performance of STEM-Females and STEM-Males under the “optimized for men” condition was high, similarly to that observed for the “neutral” and “optimized for women” conditions and was indistinguishable between them (**Figure 2A**). These results, together with those of **Table 3** and Supplementary Figure 2, suggest that academic training or related academic experiences that result in a high level of task performance and/or confidence are able to suppress the influence of the gender-related implicit associations triggered by stereotypic experimental instructions. Our results and conclusions agree with those of a previous study (Hausmann, 2014), which showed that female arts, but not female STEM or male, students, reduced their 3DMRT performance after the reactivation of gender stereotypes. Similarly, gender stereotypes reactivation promotes a reduction of math performance of female psychology, but not of female engineering, students (Crisp et al., 2009).

### Limitations and Implications

Under the different experimental conditions of the present study, academic specialization, but not the participants’ gender, was the most relevant variable to predict 3DMRT performance. Our results also reveal that the within-gender differences that derived from academic specialization (STEM vs. HUM) are larger than those observed between genders. Indeed, we only observed between-gender differences in 3DMRT performance in HUM, but not STEM, students, and these differences solely emerged in response to stereotype-reactivating experimental instructions. These findings contrast with the common belief that males have better spatial abilities than females (Devlin, 2001; Blanton et al., 2002) and with the ordinarily reported higher performance of males in mental rotation tasks in studies that specifically aim to identify “sex differences” (Linn and Petersen, 1985; Silverman and Eals, 1992; Grimshaw et al., 1995; Kempel et al., 2005; Peters et al., 2007; Silverman et al., 2007; Vuoksimaa et al., 2010; Halpern, 2013; Hyde, 2014; National Science Foundation, 2015).

At this respect, it should be noted that while we used a chronometric two-choice task, most research into sex differences in mental rotation use the pen-and-paper Mental Rotations Test (MRT) developed by Vandenberg and Kuse (1978). The MRT tends to produce larger sex differences (average *d* = 1) than chronometric tasks (average *d* = 0.3) and many studies using this second kind of procedures did not observe between genders differences (Voyer, 2011). Therefore, it might be argued that we did not observe the regularly reported gender differences because we did not use the “right” task for this. However, mental rotation chronometric tasks are as valid as psychometric tests (Voyer et al., 2006) and the MRT should not be considered as a benchmark when assessing and comparing the mental rotation abilities of males and females. In fact, the MRT does not seem to provide a pure measure of mental rotation abilities, and its singular ability to detect between gender differences might be related to the specific aspects of this test rather than to the responders’ visuospatial abilities (Kerkman et al., 2000; Voyer and Hou, 2006; Hooven et al., 2008; Bors and Vigneau, 2011). Thus, while the results obtained with either chronometric or psychometric MRTs may differ and have a limited generalizability between each other, the use of a chronometric task does not limit the validity of the results observed in the present study.

Yet, it might be argued that, because gender differences observed in mental rotation chronometric tests are small (average *d* = 0.3), our study may lack the necessary statistical power to detect them. Therefore, the results of the present study should be interpreted with caution and replicated in a larger sample of participants. However, it should be noted that, although some small effects might have failed to reach statistical significance, these power limitations did not preclude by identifying the effects of academic specialization and stereotype-reactivating experimental instructions. This hence reveals that 3DMRT performance (at least as measured in our chronometric task) is much more dependent on these factors than on the participants’ gender. Moreover, it should be also noted that the present study was not primarily intended to assess overall gender differences in visuospatial abilities but to identify a possible relationship between gender-science stereotypes and the participants performance in a specific 3DMRT task and that our study has power enough to detect even small to moderate correlations (≈ρ = 0.26 if involving all participants and ≈ρ = 0.32 for any two subgroups of participants).

In this regard, it should also be emphasized that our study did not fail to identify between-gender differences in MRT performance but showed that these differences seem to emerge under particular testing conditions and involve some, but not all, male and female participants. Yet, precisely because gender differences in 3DMRT performance depend on task and respondents’ characteristics (Sharps et al., 1994; Levine et al., 2005; Jansen-Osmann and Heil, 2007; Alexander and Evardone, 2008; Lippa et al., 2010), it might be concluded that the “sex differences” in mental rotation abilities do not arise from “sex” *per se*, but from its interaction with biographical (e.g., academic specialization) and situational variables (e.g., received instructions). In this way, our results also argue against the attempt to explain the scarce representation of women in STEM studies and professions as a result of “hardly-wired” sex differences in visuospatial and math abilities. On the contrary, our results suggest that gender socialization and stereotypes might have a larger impact in situational performance in these cognitive domains and, thereby, in shaping the perceived competence and motivation to pursuit STEM careers. These conclusions fall in line with those of other studies that have indicated an important role of females and males’ differential preferences, experiences and activities in the development of their visuospatial abilities (Flaherty, 2005; Feng et al., 2007; Sander et al., 2010; Nazareth et al., 2013; Moè, 2016). Moreover, the results and conclusions of our study also align with recent proposals which have suggested that in brain and behavior-related studies, sex and gender or, more properly, their composite resultant (sex/gender), should be considered a source of differential interactive effects with other variables rather than a binary-independent factor (Springer et al., 2012; Rippon et al., 2014; Joel and Fausto-sterling, 2016).

## Conclusion

We observed that experimental instructions might reactivate implicit biases and promote increased/decreased 3DMRT performance, but training and/or other experiences related to academic specialization moderate these effects. In this way, the present study provides evidence about when (after receiving stereotype-congruent, but not stereotype-incongruent or stereotype-nullifying instructions), how (by increasing or reducing confidence) and who (HUM, but not STEM students) might be influenced by implicit gender-science associations while performing a chronometric mental rotation task. Our results also highlight that within-gender differences might be as large as, or even bigger than, those observed between genders and, therefore, that males and females are not two uniform populations (neither in their mental rotation abilities, nor in their reaction to gender-stereotypes reactivation). Therefore, stating that “males have higher visuospatial abilities than females” is a misleading simplification that might contribute to perpetuate stereotypes. Those stereotypes and their detrimental impact on individual performance might progressively undermine the confidence and self-perceived competence of girls in cognitive domains ordinarily labeled as “masculine,” hence reducing their interest in pursuing STEM-related academic and professional careers. However, as also suggested by some results of the present study (STEM/HUM females comparison), training and positive academic experiences in those cognitive domains promote resilience against pervasive gender-science stereotypes and provide a promising avenue when trying to enhance the number of women enrolled in STEM majors.

## Author Contributions

All authors contributed to the data collection. NA programmed the experimental tasks. ÁC-G and NS contributed to manuscript editing. CF and CS-S took part in each and every step of the experiment design, implementation, data analysis/interpretation, and manuscript writing. All authors have read and approved the final manuscript.

## Conflict of Interest Statement

The authors declare that the research was conducted in the absence of any commercial or financial relationships that could be construed as a potential conflict of interest.
