# Supplementary material for: Do Gender-Related Stereotypes Affect Spatial Performance? Exploring When, How and to Whom Using a Chronometric Two-Choice Mental Rotation Task
Source: Front Psychol. 2018 Jul 24;9:1261. doi: 10.3389/fpsyg.2018.01261 (PMC6066687; doi:10.3389/fpsyg.2018.01261)
Supplement: Supplementary file 1 [file Table_1.DOCX]

**S1. Supplementary Methods.** Include Supplementary Tables 1 and 2 and Supplementary Fig. 1.

**Stimuli used in the 3DMRT.**

As mentioned in the main text, 24 stimuli from the set validated by Ganis and Kievit (2015) were taken to construct our 3DMRT. These stimuli were randomly assigned to all three experimental conditions: the “neutral”, “optimized for women” and “optimized for men” conditions. However, to make the differences among the conditions more salient, and in order to enhance the effects of the expectations’ manipulation, these stimuli were presented against a white, pink and blue background, which also included presence/absence of straight or curve stimuli-unrelated lines (see Supplementary Table 1). A pilot study proved that stimuli color and accompanying lines did not influence task performance in the absence of gender-loaded experimental instructions.


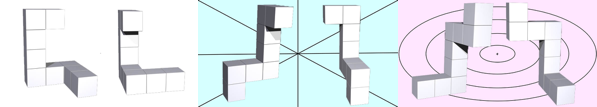


**Supplementary Figure 1.-** Left to right, examples of the stimuli and accompanying backgrounds under the “neutral”, “optimized for men” and “optimized for women” experimental conditions of the 3DMRT.

**IAT adaptation.**

As mentioned in the main text, the Gender-Science IAT script provided at the Milisecond Test Library (<http://www.millisecond.com/download/library/)> was downloaded and adapted for this study. More specifically, screen-provided instructions in the original program were faithfully translated to Spanish. Similarly, the items of the attribute lists of each original IAT script were literally translated to Spanish, but we decided to replace “biology” with “astrofísica” (astrophysics) because the general perception of biological and health sciences might be less gender-biased than that of physical sciences. We also decided to replace the general female and male nouns of the target lists with seven female and male first names, which hence makes this list more similar to that used in the original studies of this task (Greenwald et al., 1998). The original and adapted stimuli of both IATs are displayed in Supplementary Table 1.

| **IAT GENDER-SCIENCE** | | | |
| --- | --- | --- | --- |
| **Attribute A list** | | **Attribute B list** | |
| ORIGINAL | ADAPTED | ORIGINAL | ADAPTED |
| Biology* | Astrofísica* | Philosophy | Filosofía |
| Physics | Física | Humanities | Humanidades |
| Chemistry | Química | Arts | Arte |
| Math | Matemáticas | Literature | Literatura |
| Geology | Geología | English | Inglés |
| Astronomy | Astronomía | Music | Música |
| Engineering | Ingeniería | History | Historia |
|  |  |  |  |

| **Target A list** | | **Target B list** | |
| --- | --- | --- | --- |
| ORIGINAL | ADAPTED | ORIGINAL | ADAPTED |
| Man | Benjamín | Girl | María |
| Boy | Luis | Female | Alicia |
| Father | Pablo | Aunt | Ana |
| Male | Pedro | Daughter | Laura |
| Grandpa | Juan | Wife | Olga |
| Husband | José | Woman | Julia |
| Son | Daniel | Mother | Carmen |
| Uncle |  | Grandma |  |

**Supplementary Table 1.- Verbal stimuli from the original (English) and adapted (Spanish) version of the Gender-Science IAT.**

**Subjects x session distribution and gender ratios.**

As mentioned in the main text, group composition might by itself create a threatening environment for negatively stereotyped groups. Therefore, we tried to assign a similar number of participants of each experimental group to each experimental session. However, because the number of recruited participants was not the same in all groups and also because some participants did not attend the experiment, two (out of 6) experimental sessions showed skewed gender ratios (session one had a higher than desired number of males and session 4 had a higher than desired number of females). Yet, in the majority of sessions as well as in the experiment as a whole, an even proportion of females and males was achieved (see supplementary table 2)

|  | STEM-Males | STEM-Females | HUM-Males | HUM-Females | Gender ratio |
| --- | --- | --- | --- | --- | --- |
| session | (n) | (n) | (n) | (n) | (f/m) |
| 1 | 6 | 3 | 4 | 2 | 0.5 |
| 2 | 4 | 4 | 2 | 4 | 1.3 |
| 3 | 6 | 5 | 5 | 5 | 0.9 |
| 4 | 2 | 4 | 3 | 5 | 1.8 |
| 5 | 7 | 6 | 4 | 5 | 1 |
| 6 | 5 | 6 | 3 | 4 | 1.25 |
|  |  |  |  |  | Mean: 1.12 |

**Supplementary table 2.- Group participants and gender ratios at each experimental session.**
