# Supplementary material for: Do Gender-Related Stereotypes Affect Spatial Performance? Exploring When, How and to Whom Using a Chronometric Two-Choice Mental Rotation Task
Source: Front Psychol. 2018 Jul 24;9:1261. doi: 10.3389/fpsyg.2018.01261 (PMC6066687; doi:10.3389/fpsyg.2018.01261)
Supplement: Supplementary file 2 [file Table_3.DOCX]

|  | **F_5,98_** | **P** |
| --- | --- | --- |
| **Correct responses  “neutral condition”** | 1.660 | 0.167 |
| **Predicted correct responses “neutral condition”** | 0.480 | 0.822 |
| **Correct responses “optimized for men” condition** | 1.101 | 0.367 |
| **Predicted correct responses “optimized for men” condition** | 1.311 | 0.259 |
| **Correct responses “optimized for women” condition** | 0.465 | 0.832 |
| **Predicted correct responses “optimized for women” condition** | 0.541 | 0.776 |

**Supplementary Table 3.-** **Lack of effect of the experimental condition order on 3DMRT performance.** In order to control any possible practice effect that leads to artefactual changes in performance, the three experimental conditions of our 3DMRT were randomized across six experimental sessions (see the Methods section). The effectiveness of this strategy was assessed by six independent one-way ANOVAs (factor: session number) for each main dependent variable assessed in 3DMRT (objective/subjective performance x 3 experimental condition). F and p values of the independent ANOVAs when comparing the averages of the experimental sessions in the six main variables related to 3DMRT performance.
