# Supplementary material for: Do Gender-Related Stereotypes Affect Spatial Performance? Exploring When, How and to Whom Using a Chronometric Two-Choice Mental Rotation Task
Source: Front Psychol. 2018 Jul 24;9:1261. doi: 10.3389/fpsyg.2018.01261 (PMC6066687; doi:10.3389/fpsyg.2018.01261)
Supplement: Supplementary file 3 [file Table_4.DOCX]

|  | **“Neutral”** | **“Optimized for males”** | **“Optimized for females”** |
| --- | --- | --- | --- |
| **STEM-Males** | 3433.38 (843.65) | 3249.88 (755.14) | 3304.79 (642.16) |
| **HUM-Males** | 3612.11 (770.87) | 3363.61 (535.82) | 3647.92 (602.14) |
| **STEM-Females** | 3279.86 (864.37) | 3247.78 (699.97) | 3233.51 (704.39) |
| **HUM-Females** | 3460.45 (735.46) | 3376.54 (677.05) | 3438. 47 (703.58) |

**Supplementary Table 4.-** **Latency to respond (ms) by the group and experimental condition**. Values correspond to mean and standard deviation (inside brackets) for each group and experimental condition. Latencies were analyzed by a repeated measures two-way ANOVA (group x experimental condition). This ANOVA did not yield any significant effect [Group: F_3,101_=1.19, p=0.31; Experimental condition: F=0.708, p=0.402; interaction: F_3,101_=0.235 p=872].
