# Supplementary material for: Do Gender-Related Stereotypes Affect Spatial Performance? Exploring When, How and to Whom Using a Chronometric Two-Choice Mental Rotation Task
Source: Front Psychol. 2018 Jul 24;9:1261. doi: 10.3389/fpsyg.2018.01261 (PMC6066687; doi:10.3389/fpsyg.2018.01261)
Supplement: Supplementary file 6 [file Table_7.DOCX]

|  |  | **Beta** | **t** | **p value** |
| --- | --- | --- | --- | --- |
| **Included in the regression** | constant | -- | 29.88 | <0.000 |
| **model** | Academic  specialization | -0.563 | -6.85 | <0.000 |
|  | Age | 0.166 | 2.02 | 0.046 |
|  |  | **Beta** | **t** | **p value** |
| **Excluded from** | Gender | 0.055 | 0.67 | 0.50 |
| **the regression** | University major | -0.063 | -0.37 | 0.97 |
| **model** | IAT Gender-Science “influence” | 0.003 | 0.028 | 0.75 |
|  | Gender-science explicit belief | -0.112 | -1.34 | 0.183 |
|  | **Model summary** | **R** | **Adjusted R^2^** | **p value** |
|  |  | 0.568 | 0.309 | <0.001 |

**Supplementary Table 7.- Step-forward linear regression of 3DMRT performance for all the groups under the “optimized for women” condition.** In order to identify which variables included in the present study were significant predictors of participants’ objective accuracy at the “optimized for men” experimental condition, a step-forward regression analysis was conducted. All the participants were simultaneously considered and, therefore, the IAT “influence” scores (instead of the raw IAT scores), were used as possible predictors (see main text for details). As in the previous linear regression analyses, nominal variables were coded as follows: gender (males=1, females =2), academic specialization (STEM=1, HUM=2), and university major (computer sciences=1, engineering=2, journalism=3, education=4, other humanities’ studies=5).
