# Supplementary material for: Do Gender-Related Stereotypes Affect Spatial Performance? Exploring When, How and to Whom Using a Chronometric Two-Choice Mental Rotation Task
Source: Front Psychol. 2018 Jul 24;9:1261. doi: 10.3389/fpsyg.2018.01261 (PMC6066687; doi:10.3389/fpsyg.2018.01261)
Supplement: Supplementary file 8 [file Image_1.PDF]

S4. Supplementary mediation analysis results. Including Supplementary Figures 2, 3 and 4. These figures depict the results obtained after applying the same regression-based mediation analysis used in the main text and Figure 4 to rule out four alternative mediation models.

As described in the main text, the regression method for simple mediation described by Baron and Kenny [53] was used to confirm a mediatory role of confidence on the effects of implicit gender-science associations on 3DMRT observed performance. Attending to the results displayed in figure 2A, 2B and tables 3 and 4, such a mediatory role should be restricted to HUM students in the "optimized for men" condition. Thus, in order to confirm this proposed specificity, the same regression-based mediation procedure was used to test 4 alternative models.

1.- Does confidence mediates the "influence" of implicit gender-science associations on the 3DMRT observed performance of STEM students in the "optimized for men" condition?. Because no gender-related differences were found in STEM students, it might be hypothesized that, in this subset of participants, the relationship between confidence and observed performance should be independent of IAT "influence" scores. Indeed, as shown in supplementary figure 2, although confidence (expected performance) was a significant predictor of observed performance ($\beta = 0.481$, $p<0.000$), this effect was statistically independent of participants' implicit gender-science associations and IAT scores did not directly or indirectly influence 3DMRT performance in STEM students. Therefore, by showing that in another subset of participants (one at which no between-gender differences were observed) the implicit "male-science/ female humanities" association is unrelated to 3DMRT observed performance, these results confirm the specificity of our proposed model.

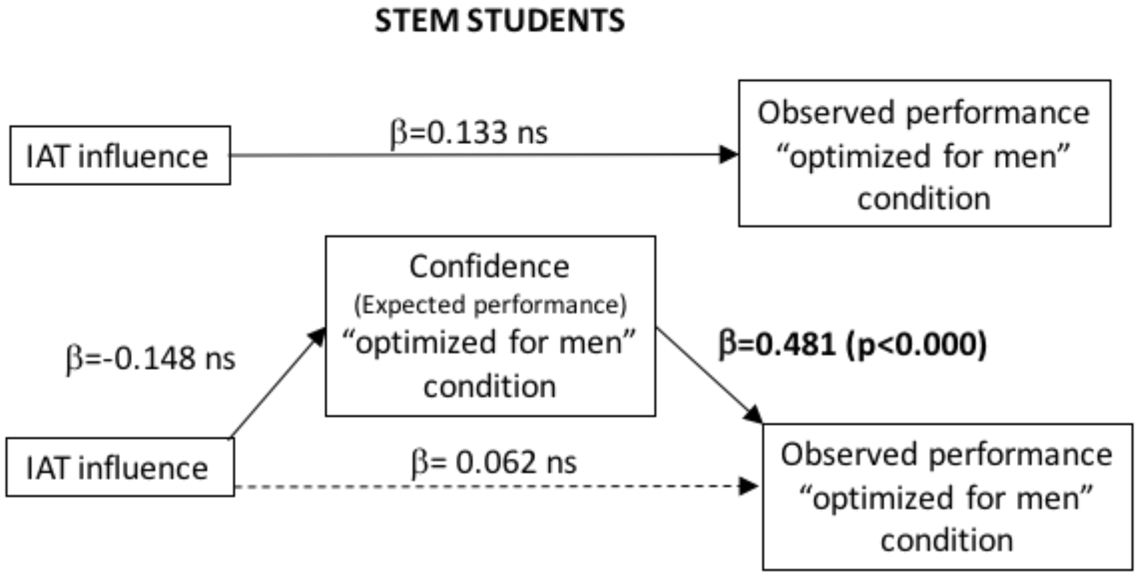

Supplementary figure 2.- The implicit "male-science/ female humanities" association does not directly or indirectly influence 3DMRT performance in STEM students.

2.- Does confidence mediates the "influence" of implicit gender-science associations on the 3DMRT observed performance of HUM students in the "neutral" condition?. Because gender-related differences between HUM-Males and HUM-

Females were restricted to the "optimized for men" condition, it might be hypothesized that the implicit "male-science/ female humanities" should not be directly or indirectly related to their observed performance at the "neutral" condition. This prediction was confirmed by the results depicted in figure 3, which also showed that the predictive value of confidence in HUM students' observed performance ($\beta = 0.51$, $p<0.000$) was statistically independent of the strength of their implicit "male-science/ female humanities" association. Again, these results confirm the specificity of our proposed model.

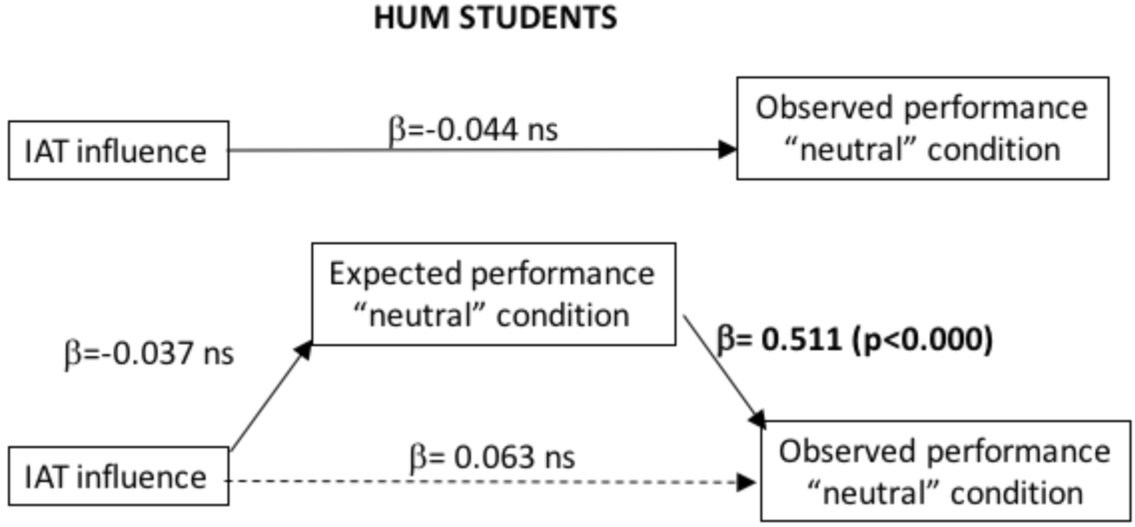

Supplementary figure 3.- The implicit "male-science/ female humanities" association does not directly or indirectly influence 3DMRT performance of HUM students in the "neutral" condition.

3 .- Does confidence mediates the "influence" of implicit gender-science associations on the 3DMRT observed performance of HUM students in the "optimized for women" condition?. Following the same rationale than in the previous case, the strength of the implicit "male-science/ female humanities" exhibited by HUM students should be neither directly or indirectly related to their observed performance at the "optimized for women" condition. As shown in supplementary figure 4, this prediction was also confirmed. These results definitively ratify that the mediatory role of confidence on the "influence" of IAT scores over 3DMRT performance is restricted to experimental conditions at which stereotype-congruent instructions are provided ("optimized for men" condition).

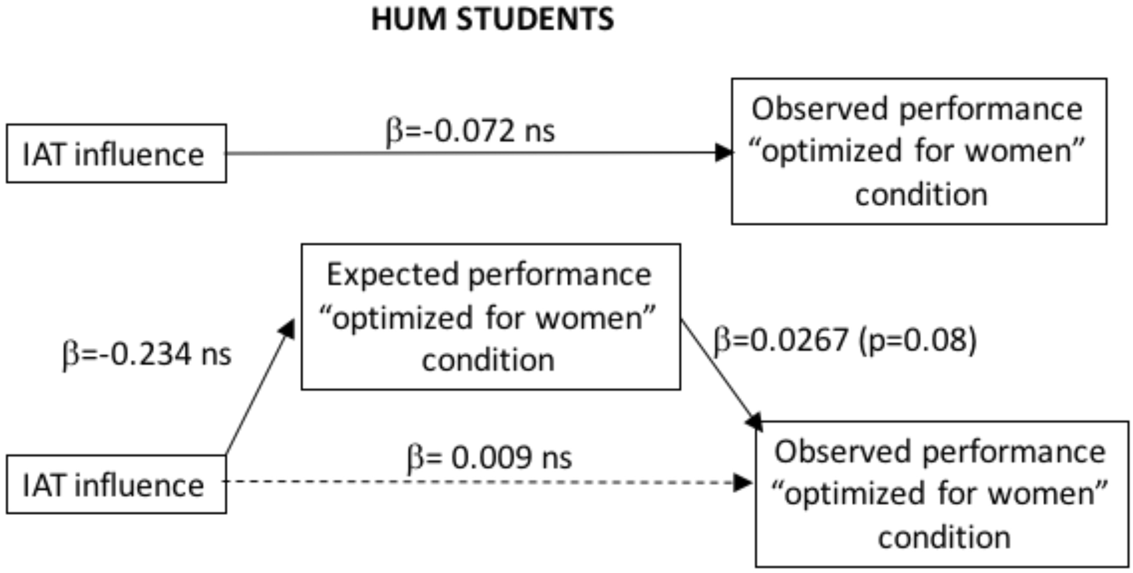

Supplementary figure 4.- The implicit "male-science/ female humanities" association does not directly or indirectly influence 3DMRT performance of HUM students in the "optimized for men" condition.

4.- Is observed performance actually mediating the "influence" of implicit gender-science associations on the 3DMRT expected performance (confidence)?. Because it was measured as a *a posteriori* expected performance index, our measure of "confidence" could actually just be a reflection of observed performance. If so, the predictive value of the IAT "influence" scores over the expected performance should entirely disappear when taking into account this putative mediating effect of observed performance. However, as shown in supplementary figure 5, this does not seem to be the case. Even when incorporated to a single regression equation that also included the observed 3DMRT performance, IAT "influence" scores did show a reduced, but still highly significant, predictive relationship towards expected performance. Thus, although there is a foreseeable relationship between expected and observed performance, the later does not determine the former and neither entirely mediates the effects of the implicit "male-science/ female humanities" association in our measure of confidence. Of note, this pattern of results is very similar to that observed by Estes and Felker (2012) [48] who, using a similar measure of confidence, showed that mental rotation performance did not mediate gender differences in confidence, but rather that gender differences in confidence strongly mediated sex differences in mental rotation performance. In this regard, our study provides converging evidence supporting a mediatory role of confidence on 3DMRT performance and also reveal that between gender differences in confidence emerge in testing conditions that reactivate an implicit "male-science/ female humanities" association.

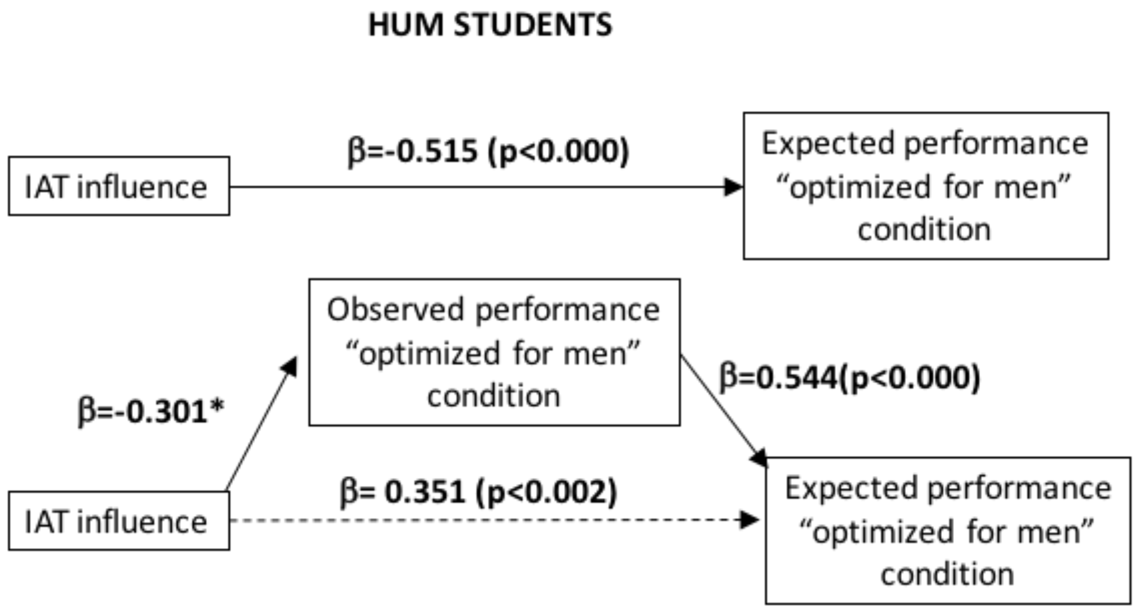

Supplementary figure 5.- The relationship between IAT influence scores and "expected performance" remains high and statistically significant after taking into account the effects of "observed performance".
